# Decreased expression of haemoglobin beta (HBB) gene in anaplastic thyroid cancer and recovory of its expression inhibits cell growth

**DOI:** 10.1038/sj.bjc.6602634

**Published:** 2005-06-14

**Authors:** M Onda, J Akaishi, S Asaka, J Okamoto, S Miyamoto, K Mizutani, A Yoshida, K Ito, M Emi

**Affiliations:** 1 Department of Molecular Biology, Institute of Gerontology, Nippon Medical School, Kawasaki, Japan; 2 Kanagawa Prefectural Cancer Center, Department of Surgery, Kanagawa, Japan; 3 Ito Hospital, Tokyo, Japan

**Keywords:** haemoglobin beta (HBB), anaplastic thyroid cancer, tumour suppressor gene, papillary thyroid cancer, 11p15.5

## Abstract

Anaplastic thyroid cancer (ATC) is one of the most fulminant and foetal diseases in human malignancies. However, the genetic alterations and carcinogenic mechanisms of ATC are still unclear. Recently, we investigated the gene expression profile of 11 anaplastic thyroid cancer cell lines (ACL) and significant decreased expression of haemoglobin beta (HBB) gene in ACL. Haemoglobin beta is located at 11p15.5, where loss of heterozygosity (LOH) was reported in various kinds of cancers, including ATC, and it has been suggested that novel tumour suppressor genes might exist in this region. In order to clarify the meaning of decreased expression of HBB in ATC, the expression status of HBB was investigated with ACL, ATC, papillary thyroid cancer (PTC) and normal human tissues. Haemoglobin beta showed significant decreased expression in ACLs and ATCs; however, in PTC, HBB expressed equal to the normal thyroid gland. In addition, HBB expressed in normal human tissues ubiquitously. To validate the tumour-suppressor function of HBB, cell growth assay was performed. Forced expression of HBB in KTA2 cell, which is a kind of ACL, significantly suppressed KTA2 growth. The mechanism of downregulation of HBB in ATC is still unclear; however, our results suggested the possibility of HBB as a novel tumour-suppressor gene.

Anaplastic thyroid cancer is one of the most fulminant human malignancies, with a mean survival time among patients of less than 1 year after diagnosis, regardless of treatment (Passler *et al*, 1999; Voutilainen *et al*, 1999). It is considered that anaplastic thyroid cancer (ATC) is thought to arise mainly from a background of differentiated (papillary or follicular) cancer, on the basis of clinicopathological observations that ATC is often accompanied by such cells and that anaplastic tumours tend to arise in patients who had previously been treated for differentiated cancer of the thyroid (Nakamura *et al*, 1992; Kitamura *et al*, 2000a). Little is known about the genetic alterations in anaplastic thyroid cancer; only TP53 (Kitamura *et al*, 2000b) and beta-catenin (Garcia-Rostan *et al*, 1999) mutations have been discovered in ATC.

Recently, we investigated the gene expression profile of anaplastic thyroid cancer cell lines (ACLs) with cDNA microarray analysis (Onda *et al*, 2004a) and found the novel gene alterations that related to ATC/ACL carcinogenesis. The results of microarray analysis showed that haemoglobin beta (HBB) gene significantly decreased expression in ACLs, compared to the expression of normal thyroid gland. (Data will be provided upon request to the corresponding author.)

Haemoglobin beta locates at 11p15.5 and its main function is oxygen transporter activity (Thein *et al*, 1990). The normal adult haemoglobin (HbA) tetramer consists of two alpha chains and two beta chains (Yasuda *et al*, 2002), and it is known that mutant HBB causes sickle cell anaemia (Persons, 2003). Loss of heterozygosity (LOH) of this locus has been reported in ovarian cancer (Launonen *et al*, 2000), glioma (Schiebe *et al*, 2001), breast cancer (Xu *et al*, 2001; Roy *et al*, 2003), lung cancer (Xu *et al*, 2001) and paediatric thyroid carcinoma (Pauws *et al*, 2001). In addition, our previous report showed up to 33% of LOH at 11p15 (D11S922) in anaplastic thyroid cancer (Kitamura *et al*, 2000a). Considering these facts, it is fascinating for us to investigate the relation between decreased expression of HBB and ACL/ATC progression.

In this study, we examined the expression status of HBB in ACLs, primary ATC and several normal human tissues with the reverse transcriptase–PCR (RT–PCR) method. In addition, protein expression was investigated in ATC, papillary thyroid cancer (PTC), follicular thyroid cancer (FTC), adenoma (FAD) and chronic thyroiditis (CTH) using the immunohistochemical method, because, in some cases, it is considered that ATC might derive from PTC components (Nakamura *et al*, 1992; Ozaki *et al*, 1999; Kitamura *et al*, 2000a). Next, the efficacy of the overexpression of HBB to cell growth in KTA2, which is an ACL and where no expression of HBB was confirmed, was evaluated.

## MATERIALS AND METHODS

### Anaplastic thyroid cancer cell lines

In all, 11 cell lines that were derived from ATC, 8305c, 8505c, ARO, FRO, TTA1, TTA2, TTA3, KTA1, KTA2, KTA3 and KTA4 were used for this study. The 8305c and 8505c cell lines were maintained in Dulbecco's modified Eagle medium (Invitrogen, Carlsbad, CA, USA), ARO and FRO cell lines in minimum essential medium, and the other seven lines in RPMI 1640. All media contained 10% foetal bovine serum but no antibiotics. The cells were cultured in a 37°C incubator under 5% CO_2_ atmosphere.

### Patients and specimens

Primary ATC, PTC and noncancerous thyroid gland were excised from 10 patients who underwent surgery at the Ito Hospital, Tokyo; the samples were frozen immediately and stored at −80°C. All patients had given informed consent according to guidelines approved by the Institutional Research Board. All tumour specimens that we analysed contained more than 70% tumour cells.

For immunohistochemical analysis, 14 archived paraffin-embedded samples of ATC and 15 PTC samples were used.

### RNA extraction and cDNA synthesis

Extraction of RNA from cell lines and tissues was performed as described previously (Onda *et al*, 2004a, 2004b). Using 5 *μ*g of RNA as a template, cDNAs were synthesised in the usual manner. Briefly, the template was mixed with 1 *μ*l of oligo dT_12–18_ (Invitrogen, Carlsbad, CA, USA), used for annealing primer and denatured at 70°C for 10 min. Then, 200 U of Reverse Transcriptase II (Wako Pure Chemical, Tokyo, Japan), reaction buffer, 40 U of RNase inhibitor (Wako Pure Chemical) and 10 *μ*mol dNTPs were added. This mixture was incubated at 42°C for 60 min, after which the product was treated with 2 U of RNase (Wako pure chemical) at 37°C for 20 min.

### Semiquantitative RT–PCR

In order to evaluate the expression of HBB in ACL, ATC, PTC and normal human tissues, semiquantitative RT–PCR (SQ–PCR) was performed. For the comparison, five normal thyroid samples were served as a control in SQ-PCR. To adjust the amount of transcribed cDNA, GAPDH was selected as an internal control and SQ-PCR experiments were carried out as previously described, after adjustment of the amount of template cDNA (Onda *et al*, 2004a). The primer sequences for GAPDH were 5′-ggaaggtgaaggtcggagt-3′ (forward) and 5′-tgggtggaatcatattggaa-3′ (reverse). Sequence information was collected from the NCBI GenBank (http://www.ncbi.nlm.nih.gov/), and primers for HBB were designed with Primer 3 software (http://www-genome.wi.mit.edu/cgi-bin/primer/primer3_www.cgi). The HBB forward primer was 5′-ggagatgcctcagaaactgc-3′ and the reverse primer was 5′-aggttggaggtcggaaagtt-3′. SQ-PCR experiments were performed with 1 *μ*l of cDNA for template, 5 U of Takara EX Taq (Takara, Otsu, Japan), 1 × PCR buffer (10 mM Tris-HCl, 50 mM KCl, 1.5 mM MgCl_2_) and reverse primers in 30 *μ*l of total reaction mixture. PCR conditions for each gene were optimised in their respective linear phases of amplification.

For the evaluation of differences of HBB expression between ACL, ATC, PTC and normal thyroid gland, 10 *μ*l of each SQ-PCR product was electrophoresed on a 2.0% agarose gel and stained with ethidium bromide. After staining, the density of each sample spot was measured by AlphaImager 3300 (AlphaIonotech, San Leandro, CA, USA) with background revision. All SQ-PCR experiments were duplicated.

### Quantitative RT–PCR

In order to quantify the expression of HBB in ACL, ATC, PTC and exogenous expression in cell growth assay, real-time quantitative RT–PCR (Q-PCR) was performed. Q-PCR was carried with qPCR Mastermix for Syber Green I (Eurogenetec, Seraing, Belgium) and ABI 7700 (Applied Biosystems, Foster City, CA, USA) described previously (Onda *et al*, 2004b). Primers for Q-PCR of HBB were the same as those used in the SQ-PCR study. The template of Q-PCR was the same as that used in SQ-PCR, where the amount of cDNA monitoring of *GAPDH* expression was adjusted.

The expression difference between normal thyroid tissue and sample X in ACL, ATH and PTC is defined as follows: 
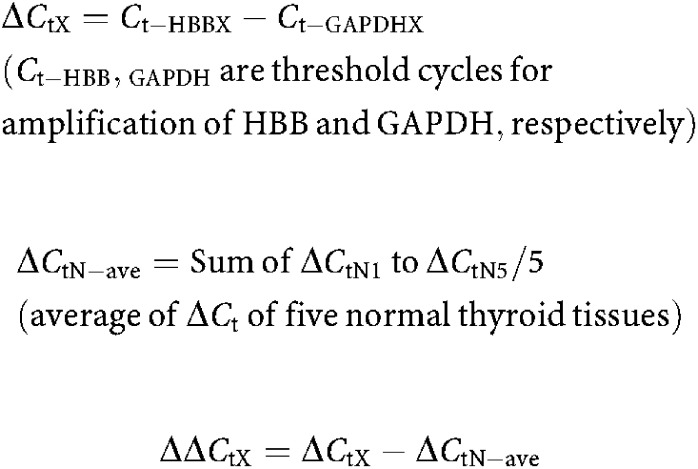


Expression ratio of HBB to normal thyroid tissue (Sample X/average of five normal thyroid tissues)=2^(−ΔΔCt X)^

### Immunohistochemical analysis

To determine protein expression of HBB, we performed immunohistochemical analyses using 14 paraffin-embedded samples of primary ATC and 15 PTC samples. ATC and PTC samples were collected at the Ito Hospital, Tokyo, Japan. In addition, for the evaluation of HBB expression in FTC, FAD and CTH, AccuMax thyroid cancer array and thyroid disease array (ISU ABXIS Co., Ltd, Seoul, South Korea) were applied. Anti-HBB antibody was used (sc-21757, Santa Cruz Biotechnology, Inc., Santa Cruz, CA, USA) with 1 : 100 dilutions. Antigens were microwaved prior to immunostaining with VECTASTAIN Elite ABC kits (Vector Laboratories Inc., Burlingame, CA, USA) and Dako ENVISION kits (Dako corporation, Carpinteria, CA, USA) following the manufacturers' instructions. The sections were counterstained with haematoxylin, and then scanned at low power to identify areas that were evenly stained. Estimates of the numbers of positive cells were scored as follows: negative, 0%; 1, 1–10%; 2, 11–25%; 3, 26–50%; 4, >50% positive (Saiz *et al*, 2002). Two independent investigators performed the estimation.

### Construction of HBB expression vector

The expression vector of HBB was constructed with pcDNA3.1 directional Expression Kit (Invitrogen, Carlsbad, CA, USA) following the manufacturer's instruction with some modifications. First, to generate a full length of the coding sequence of HBB, PCR was performed using cDNA that was reverse transcribed from pooled human placenta RNA. The cloning forward primer was 5′-caccatggtgcatctgactcc-3′, and reverse primer was 5′-gtgatacttgtgggccagggc-3′. PCR condition was the same as that used in SQ-PCR. This reaction generated a 443 bp PCR product that contained CACC sequence in front of the ATG start codon and deleted TAG stop codon from the original HBB sequence. The PCR product was electrophoresed with 2.0% NuSieve GTG agarose (Cambrex Bio Science Rockland Inc., Rockland, ME, USA) and stained with ethidium bromide. The proper size of the PCR product was excised and nucleic acid extracted with QIAquick Gel Extraction kit (QIAGEN, Tokyo, Japan). In all, 4 *μ*l of gel-extracted PCR product was ligated into pcDNA3.1 expression vector with 30 min incubation at room temperature. The ligated plasmid was mixed with 100 *μ*l of one-shot chemically competent *Escherichia coli* (Invitrogen) and incubated on ice for 30 min, and then it was heat shocked at 42°C for 30 s and placed on ice immediately. Transformed *E. coli* was incubated in 300 *μ*l of SOC medium (Invitrogen) at 37°C in the orbital shaker, and then 100 *μ*l of cultured SOC medium was plated on the LB plate containing 100 *μ*g ml^−1^ ampicillin and it was incubated at 37°C for overnight. Several colonies were selected and sequences confirmed in the usual manner. Proper clone was incubated in LB medium at 37°C overnight and plasmid DNA was extracted using QIAfilter Plasmid Midi Kit (QIAGEN).

### Transfection of HBB to KTA2 cell line and the evaluation of the exogenous expression of HBB

In order to evaluate the effect of HBB in anaplastic thyroid cancer, HBB expression vector, pcDNA HBB expression vector was transfected into KTA2, which did not express HBB originally. The day before transfection, 1 × 10^3^ of KTA2 cells were plated in a 24-well plate and cultured with the same condition as described previously. After 24 h, the cells reached 60–70% confluence. Prior to transfection, the medium was changed to fresh RPMI1640. In all, 200 ng of pcDNA HBB was mixed up to 50 *μ*l of Opti-MEM I medium (Invitrogen) and siPORT XP-1 (Ambion, Austin, TX, USA) subsequent to XP-1's instructions to make pcDNA HBB/XP-1 complex. The complex was transfected into arranged KTA2 and it was cultured up to 7 days. At the determined time points of days 0, 3, 5 and 7, cells were collected and RNA was extracted with TRIzol (Invitrogen, Carlsbad, CA, USA) (Onda *et al*, 2004b). The exogenous expression of HBB was confirmed with both SQ-PCR and Q-PCR in the same manner as described previously.

### Cell growth assay

To determine the influence of gene expression of HBB, cell growth assay (Smith *et al*, 2003) was carried out. In total, 3000 KTA2 cells were plated on a 24-well plate and, after 24 h, 200 ng of pcDNA HBB plasmid was transfected. Cells were fixed with 10% formalin for 10 min at the determined time points, 0, 3, 5 and 7 days after transfection. After washing two times in water, fixed cells were stained with 0.1% crystal violet for 10 min, and then washed three times with water to remove the excess crystal violet solution. The stained crystal violet was eluted with 200 *μ*l of 10% acetic acid and the absorbance of 590 nm was measured with a spectrophotometer. The difference of cell growth was calculated using Student's *t*-test. This statistical procedure was performed by Statview version 5.0 (SAS Institute Inc., Cary, NC, USA). The experiments were triplicated.

### Single-strand conformation polymorphism (SSCP) analysis of HBB gene

In order to analyse mutation in the HBB gene, PCR-SSCP analysis was carried out (Onda *et al*, 1997; Harada *et al*, 2002). Genomic DNA was extracted from 11 ACLs in a usual manner. With regard to the entire coding region of HBB, the first exon to the third exon were examined. The primers used for SSCP were as follows: (exon 1) 1F: 5′-agcaacctcaaacagacacc-3′, 1R: 5′-gtctccacatgcccagtttc-3′, (exon 2) 2F: 5′-ttggtctattttcccaccctta-3′, 2R: 5′-tcaagcgtcccatagactca-3′, (exon 3) 3F: 5′-cctcttatcttcctcccacag-3′, 3R: 5′-gatgctcaaggcccttcata-3′. These primers were designed to cover the entire exon of the HBB gene. Heat-denatured PCR product was electrophoresed on a 6% long ranger gel (Cambrex, East Rutherford, NJ, USA) with 5% glycerol, 5 W for 4 h. DNA fragments were visualised by silver staining using the Plus One DNA silver-staining kit (Pharmacia Biotech, Tokyo, Japan) following the manufacturer's manual.

### Methylation-specific PCR in the promoter legion of the HBB gene

In order to evaluate the methylation status of the promoter region of the HBB gene, methylation-specific PCR analysis (MSP) was performed. Genomic DNA from 11 ACLs was bisulphite modificated using the CpGenome DNA modification kit (CHEMICOM International, Inc., Temecula, CA, USA) following the manufacturer's instruction. Sequence information of the promoter region of the HBB gene was downloaded from UCSC genome browser (http://genome.cse.ucsc.edu/cgi-bin/hgGateway) and primers for MSP were designed with Methprimer program (http://www.ucsf.edu/urogene/methprimer/index1.html). Primers used for MSP are as follows: for methylated DNA (M), forward: 5′-ttagaagagttaaggataggtacgg-3′; reverse: 5′-taccccacaaaacaataacgac-3′; for unmethylated DNA (UM), forward: 5′-ttagaagagttaaggataggtatgg-3′; reverse: 5′-cttaccccacaaaacaataacaac-3′. The size of PCR products was 250 and 252 bp.

## RESULTS

### Expression of HBB in ACLs, primary ATC and PTC

**Figure 1:**
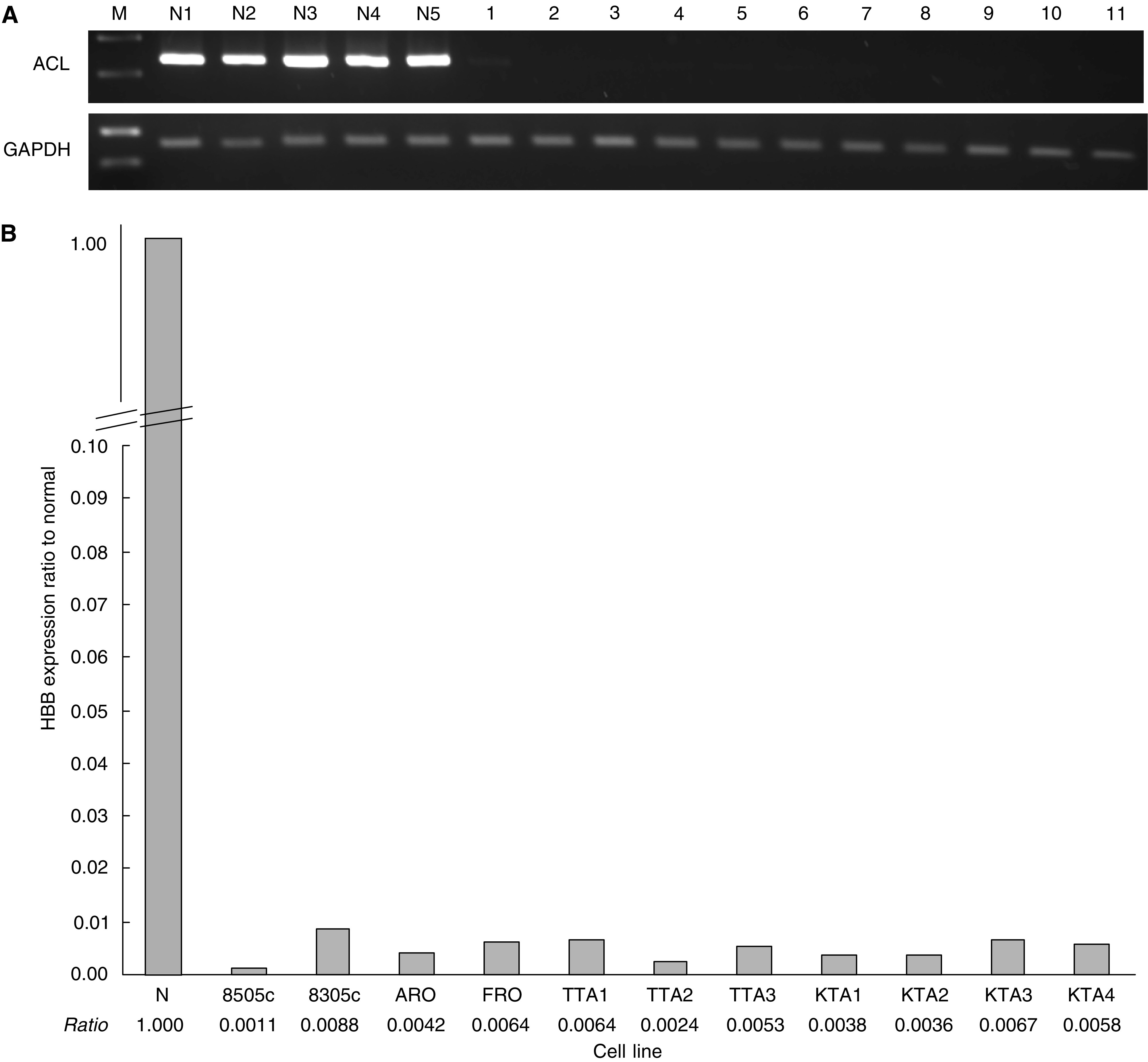
(**A**) Expression of HBB in ACLs. Compared to five normal thyroid gland expressions, HBB expression was significantly decreased in ACL samples. M: size marker, N1–5: normal thyroid, 1: 8305c, 2: 8505c, 3: ARO, 4: FRO, 5: TTA1, 6: TTA2, 7: TTA3, 8: KTA1, 9: KTA2, 10: KTA3, 11: KTA4. (**B**) Result of Q-PCR. The average expression of five normal thyroid glands was settled as 1.00, and relative expression ratio compared to normal was calculated. Average expression of HBB in ACL was below 1% of normal thyroid gland.

In order to evaluate the expression of HBB in ACLs, SQ-PCR and Q-PCR were performed. Compared to normal thyroid glands that were derived from five PTC patients, expression of HBB was significantly decreased in 11 ACLs (Figure 1A and B). Average expression of HBB in ACL was below 1% of the average expression in a normal thyroid gland.

**Figure 2:**
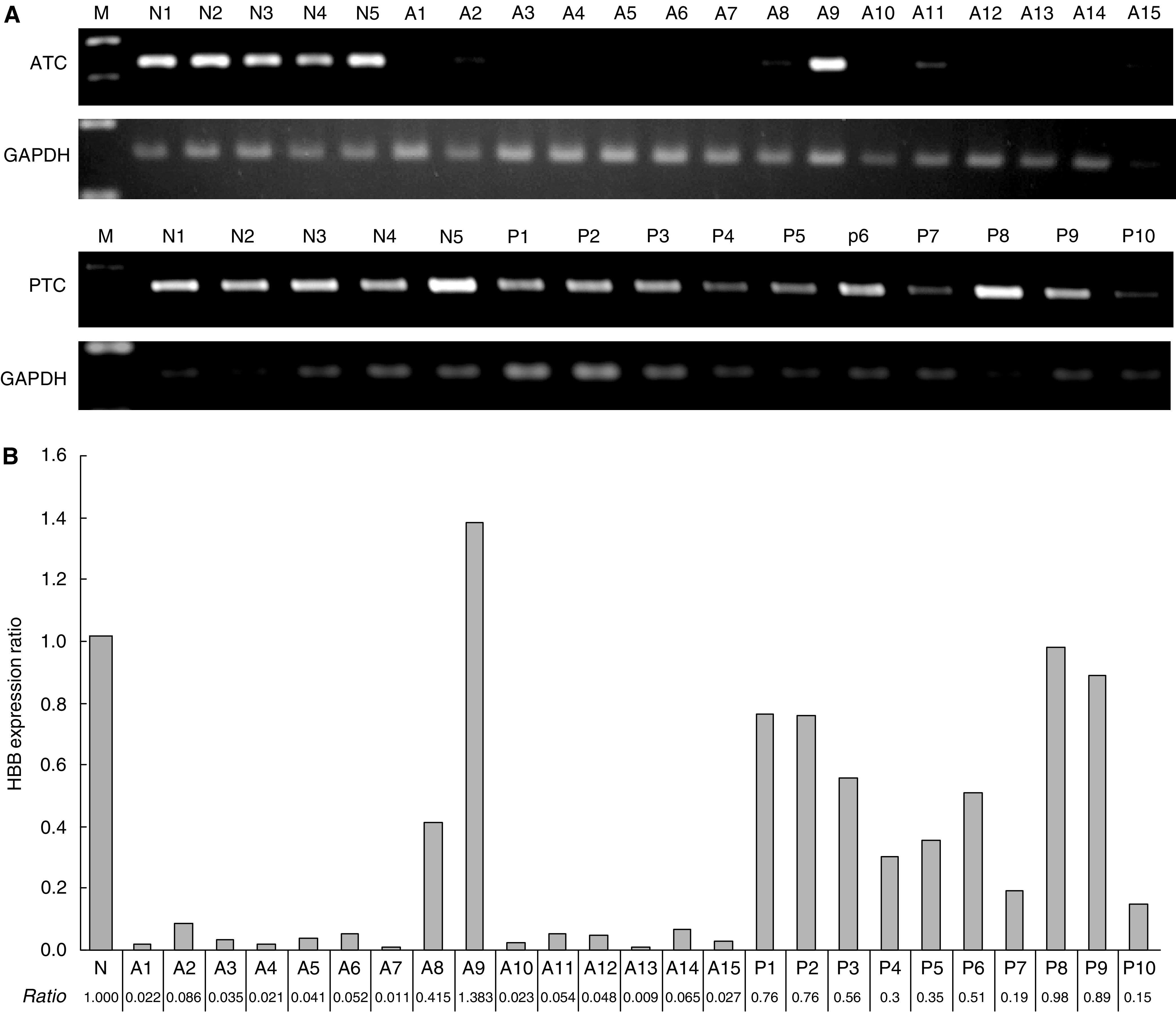
(**A**) Expression of HBB in primary thyroid cancer by SQ-PCR. In primary ATC samples, HBB expression was decreased compared to normal thyroid gland. On the contrary, HBB expression was almost equal to the expression of normal thyroid glands. M: size marker, N1–5: normal thyroid, A1–15, primary anaplastic thyroid cancer samples (ATC), P1–10, primary PTC samples. (**B**) Relative expression of HBB to normal thyroid gland is presented. Haemoglobin beta expression of normal thyroid is the average of five normal thyroid glands. When the expression of normal thyroid was settled as 1.00, the ratio represented relative expression of HBB in the sample.

Then, HBB expression was assessed with ATC and PTC samples. In ATC, the expression of HBB was significantly decreased as seen in ACLs, except in one case (A9), with SQ-PCR and Q-PCR assay. Haemoglobin beta expression in ATC was 0.9–8.6% of normal thyroid gland expression, besides A8 and A9 cases. On the other hand, in PTC samples, expression of HBB was 15–98% of normal thyroid. Even in PTC cases, decreased HBB expression was observed; however, the expression of HBB in PTC was higher than that in ATC (Figure 2A and B).

**Figure 3:**
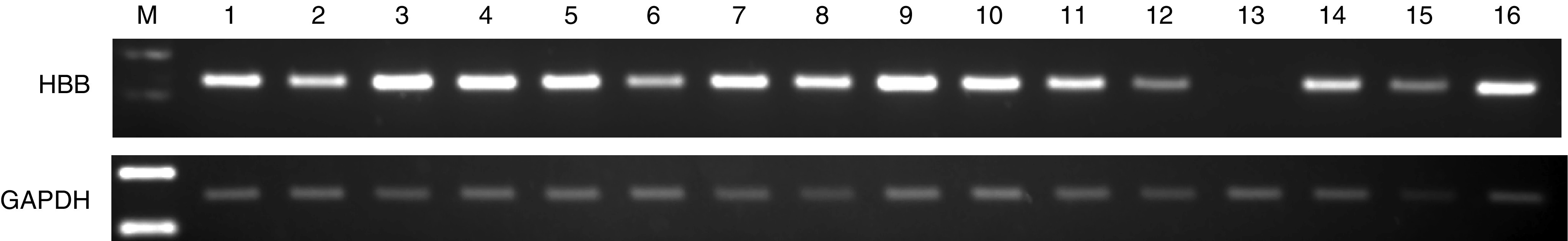
Expression of HBB in 16 normal human tissues. Haemoglobin beta was expressed ubiquitously except in the ovary. M, size marker, 1. Heart, 2. Whole brain, 3. Placenta, 4. Lung, 5. Liver, 6. Skeletal muscle, 7. Kidney, 8. Pancreas, 9. Spleen, 10. Thymus, 11. Prostate, 12. Testis, 13. Ovary, 14. Small intestine, 15. Colon, 16. Leukocyte.

To validate the expression of HBB in other human normal tissues, SQ-PCR was achieved with 16 human normal tissues. As shown in Figure 3, HBB was expressed in all tissues, besides the ovary, with various intensities. These results presented that HBB was expressed ubiquitously in normal human tissues including the thyroid gland.

### Construction of HBB expression vector and forced expression in KTA2 cell

**Figure 4:**
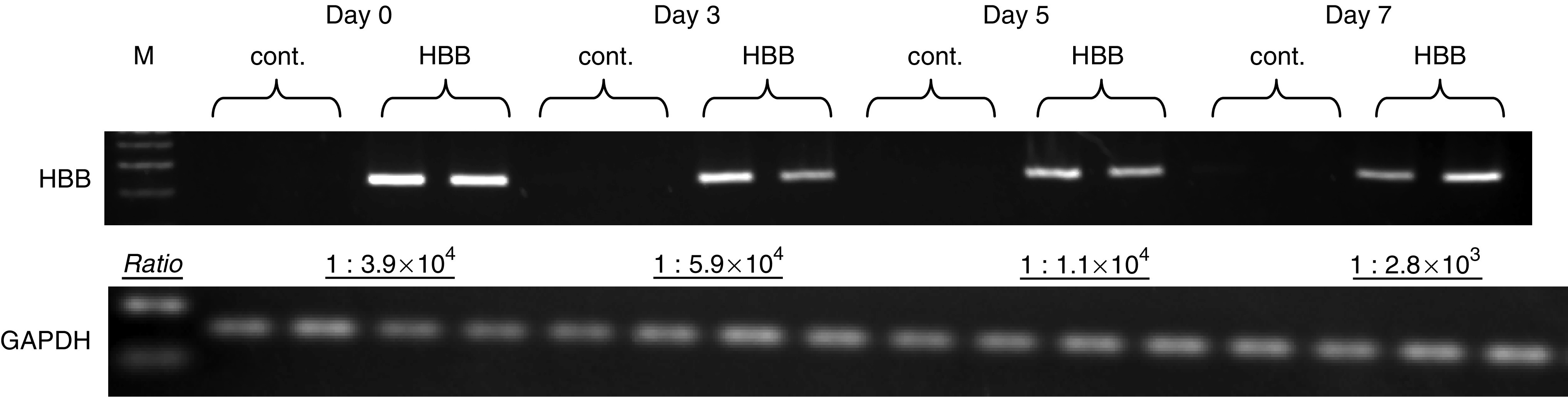
Upregulated expression of HBB at KTA2 with pcDNA HBB vector. Compared to control (pcDNA empty vector), transfected cells with pcDNA HBB expression vector showed upregulated expression of HBB at the determined points of days 0, 3, 5 and 7 from the transfection. M, size marker, cont., control, pcDNA empty vector, HBB, pcDNA HBB-transfected samples. Ratio, Exogenous expression of HBB against control in each time points, measured by Q-PCR.

Using pcDNA 3.1 plasmid, HBB expression vector, namely pcDNA HBB, was developed. To confirm forced expression of HBB, pcDNA HBB was transfected into KTA2 and RNA was collected at days 0, 3, 5 and 7. Exogenous expression of HBB within transfected KTA2 cell line was confirmed with SQ-PCR and Q-PCR analysis (Figure 4). Compared to the control, HBB expression was upregulated in transfected KTA2.

### Cell growth assays

**Figure 5:**
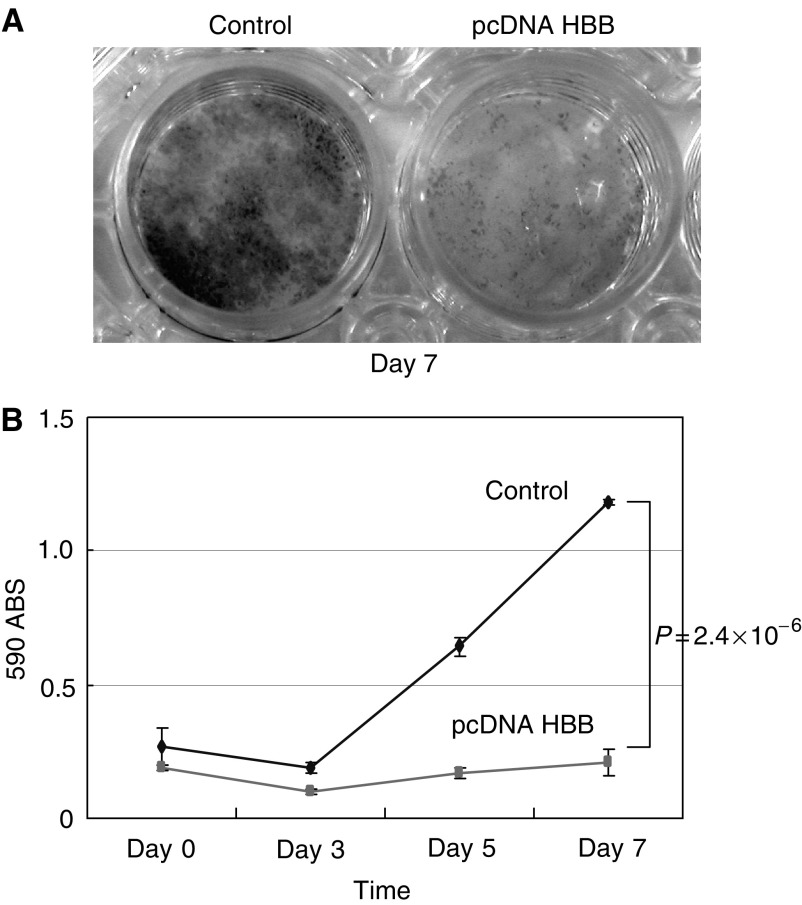
The results of cell growth assay. (**A**) Representative image of KTA2 staining with crystal violet. The cells were stained at 7 days after transfection. Control, transfected KTA2 with pcDNA empty vector, HBB, transfected KTA2 with pcDNA HBB expression vector. The experiments were triplicated. (**B**) Graphical view of KTA2 cell growth. Compared to control, pcDNA HBB transfected cell showed growth suppression (*P*=2.4 × 10^−6^, at the point of day 7, *t*-test).

In order to clarify the effect of HBB in cell growth, cell growth assay was performed. KTA2 cells that were transfected pcDNA HBB showed the suppression of cell growth, compared to the control (Figure 5A). Haemoglobin beta-transfected cells showed a slow-growing curve (Figure 5B), and, at the point of day 7, the difference of cell growth between pcDNA HBB-transfected cells and pcDNA empty vector-transfected cells (control) reached statistical significance (*P*=*2.4 × 10*^−*6*^).

### Immunohistochemical analysis of HBB expression with ATC, PTC, FTC, FAD and CTH

**Figure 6:**
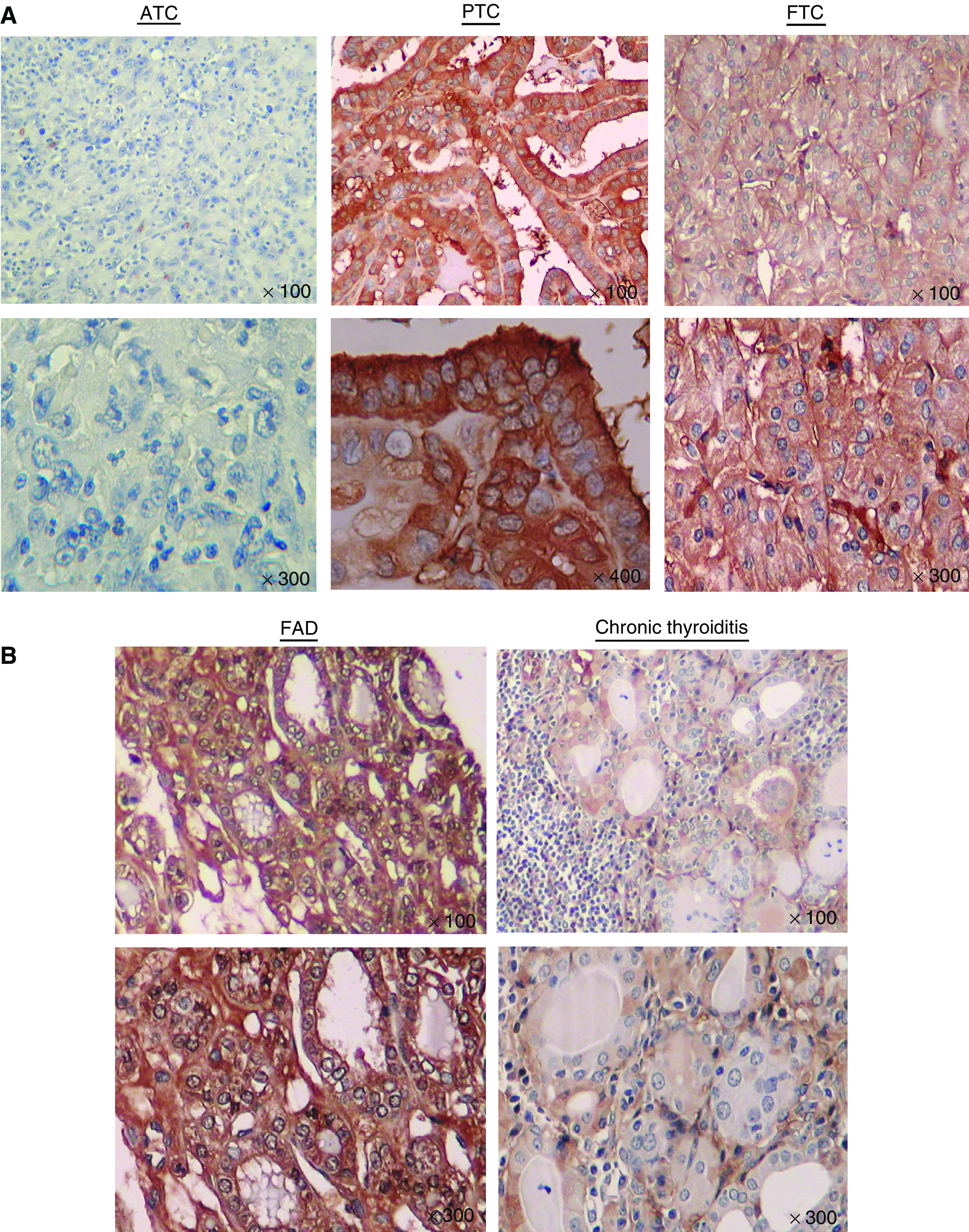
Immunohistochemical analysis of HBB expression. (**A**) Little expression of HBB was confirmed in ATC samples. On the contrary, HBB expressed in PTC and FTC, mainly stained in cytoplasm. Upper: × 100, lower: × 300 and × 400. (**B**) Haemoglobin beta expressed in FDA and CTH. However, less staining was seen in CTH, compared to other histological types.

To evaluate HBB protein expression, immunohistochemical analysis was performed with 14 primary ATC and 15 PTC samples. In addition, using tissue microarray, nine FTC, five FAD and five CTH samples were assessed. In PTC, HBB expression was confirmed with strong intensity and the average score was 2.53. On the contrary, weak expression was seen in ATC samples (Figure 6A), and the average score was 0.71 (Table 1). In FTC, FAD and CTH, stronger expression of HBB was seen compared to ATC samples (Figure 6A and B), and the average staining scores were 3.33, 2.8 and 1.6, respectively.

### Mutation analysis of HBB gene

**Figure 7:**
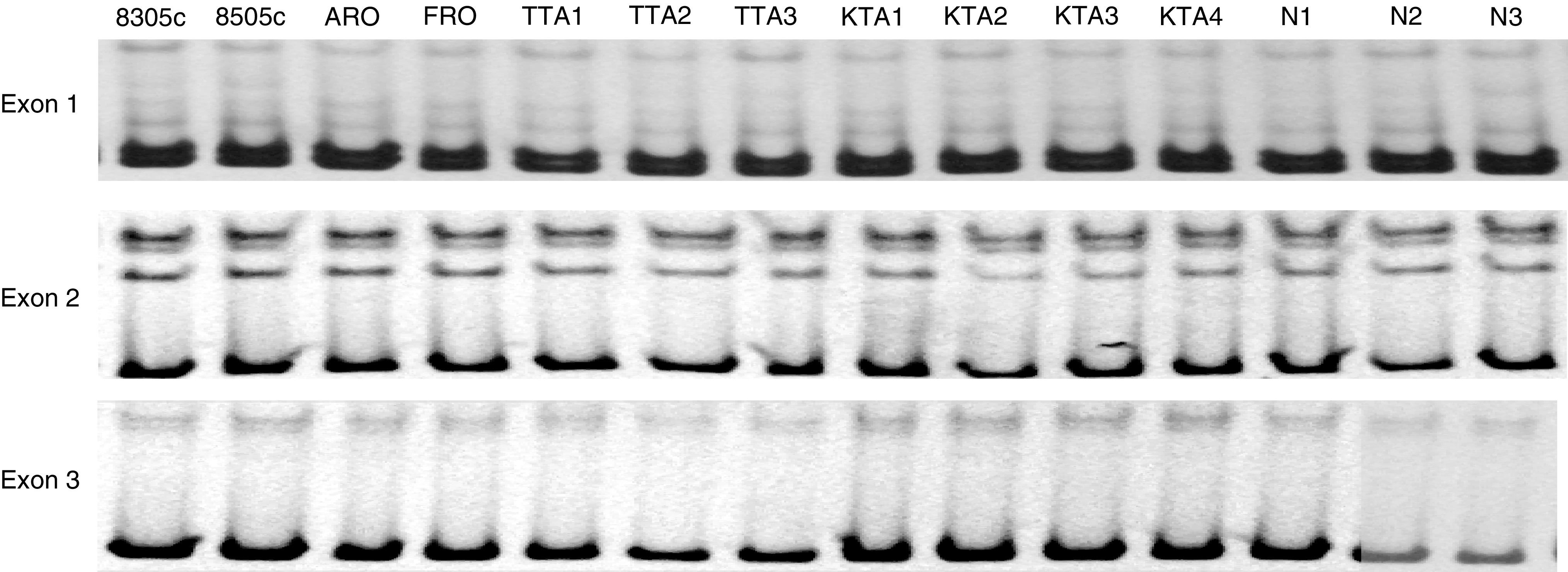
Results of SSCP analysis of HBB gene with 11 ACLs and three normal thyroid glands. Within coding region from exon 1 to 3, no mutation was confirmed.

In order to detect the mutation of HBB gene, SSCP analysis was performed using genomic DNA from 11 ACLs. No mutation from exon 1 to 3 was seen in 11 ACL samples (Figure 7).

### Methylation-specific PCR

**Figure 8:**
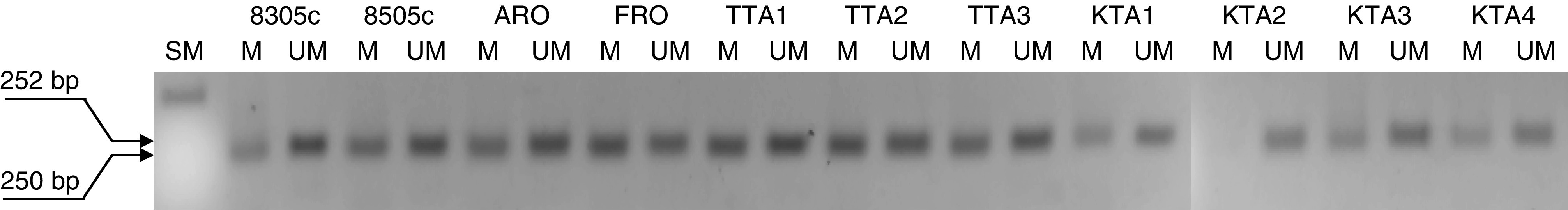
Results of MSP analysis within the promoter region of HBB gene with 11 ACLs. Generally speaking, all ACL were hemimethylated in the promoter region except KTA2. Only KTA2 was not methylated in this region.

To evaluate the methylation status of the HBB promoter region, MSP assay was carried out with 11 ACL. Within 10 cell lines except KTA2, PCR product was seen both in M and UM primer sets. Only KTA2 was not methylated in the promoter region. This result showed that the promoter region of HBB gene was mainly hemimethylated and it suggested that methylation was one of the reasons of downregulation of HBB expression in anaplastic thyroid cancer (Figure 8).

## DISCUSSION

Haemoglobin beta is one of the globins chain components of haemoglobin A, whose basic function is oxygen transport (Giardina *et al*, 1995). There are several reasons for the reduction of HBB expression; for example, partial deletion of the terminal portion of HBB gene (Orkin *et al*, 1979), nonsense mutation (Chang and Kan, 1979) and frameshift deletion (Orkin and Goff, 1981) cause beta-thalassemia.

Genetically, HBB locates at 11p15.5 (Bepler *et al*, 1999) and this locus contains embryonic and adult beta-like globin genes that are ordered as they are expressed during development. High-level expression of the *beta-globin* gene cluster is regulated by the locus control region (LCR) (Sawado *et al*, 2003). Also, at this locus, loss of imprinting (LOI) had been reported in head and neck squamous cell cancers (Rainho *et al*, 2001). Thus, chromosomal location of 11p15.5 is interesting from the viewpoint of human genetics. In addition, there were several reports of LOH in various kinds of cancers at this locus. In this region, there were two distinct tumour-suppressor loci that implicated breast cancer progression and metastasis (Karnik *et al*, 1998). It is suggested that an LOH of 11p15.5 might relate to poor prognosis of nonsmall-cell lung cancer (Bepler *et al*, 2002). We previously reported up to 33% LOH at this locus in primary ATC (Kitamura *et al*, 2000a). Considering these results, it is highly suggested that novel tumour suppressor genes might be exist at this locus.

In this report, we showed the significant decreased expression of HBB in ACL and ATC samples. This is the first report of decreased expression of HBB in anaplastic thyroid cancer. On the other hand, HBB expression was seen in normal thyroid gland, PTC, FTC, FAD and CTH samples. In addition, expression of HBB was observed in other normal human tissue ubiquitously.

Generally speaking, it is considered that a large part of ATC arises from differentiated thyroid cancer with anaplastic transformation (Hunt *et al*, 2003). Thus, our results suggest the possibility that loss of expression HBB might relate to anaplastic transformation of differentiated thyroid cancer.

To clarify the role of HBB in cancer cell growth, cell growth assay was performed with ACL KTA2, which has no expression of HBB basically. Interestingly, exogenous expression of HBB suppressed the cell growth significantly. Our results presented the possibility of the tumour suppressor activity of HBB. There are several reasons for gene silencing, including LOH and methylation of promoter regions. In this study, LOH was not examined because normal-tumour paired DNA was not available. However, methylation-specific PCR in ACLs revealed the hemimethylated status of the promoter region of the HBB gene. With regard to mutation search, there was no mutation in the coding region of the HBB gene. Considering these results, it was suggested that methylation of the promoter region was one of the reasons for the downregulation of the HBB gene. However, we could not detect promoter methylation in KTA2 cell; so it is also suggested that there might be other mechanisms besides promoter methylation for the downregulation of HBB expression in anaplastic thyroid cancer.

Haemoglobin beta is one of the well-investigated genes in the field of blood diseases. However, nobody paid attention to this gene in the field of ‘cancer’. Even physiologically, HBB expression is controlled intricately (Stamatoyannopoulos, 2005). In the viewpoint of cancer genetics, it strongly required further examination of the mechanism of the downregulation of HBB in anaplastic thyroid cancer.

## Figures and Tables

**Table 1:** Score of HBB expression with immunostaining method

**ATC**		**PTC**		**FTC**		**FAD**		**CTH**	
**Case**	**Score^a^**	**Case**	**Score^a^**	**Case**	**Score^a^**	**Case**	**Score^a^**	**Case**	**Score^a^**
AT-1	1	PT-1	1	D5	3	F4	2	B5	2
AT-2	0	PT-2	3	E1	4	F6	1	C1	1
AT-3	1	PT-3	4	E3	3	G2	4	C3	2
AT-4	0	PT-4	4	F1	4	G4	3	C5	2
AT-5	0	PT-5	2	G1	4	G6	4	D1	1
AT-6	0	PT-6	1	G3	4				
AT-7	2	PT-7	4	G5	2				
AT-8	0	PT-8	2	F7	4				
AT-9	1	PT-9	3	F9	2				
AT-10	0	PT-10	1						
AT-11	1	PT-11	2						
AT-12	2	PT-12	3						
AT-13	2	PT-13	4						
AT-14	0	PT-14	3						
		PT-15	1						

Ave.	0.71	Ave.	2.53	Ave.	3.33	Ave.	2.8	Ave.	1.6

a Estimates of the numbers of positive cells; negative, 0%, 1, 1–10%; 2, 11–25%; 3, 26–50%; 4, >50% positive.
